# Use of flow cytometry for high-throughput cell population estimates in brain tissue

**DOI:** 10.3389/fnana.2012.00027

**Published:** 2012-07-11

**Authors:** Nicole A. Young, David K. Flaherty, David C. Airey, Peter Varlan, Feyi Aworunse, Jon H. Kaas, Christine E. Collins

**Affiliations:** ^1^Department of Psychology, Vanderbilt University, NashvilleTN, USA; ^2^Vanderbilt University Medical Center, Flow Cytometry Shared Resource, NashvilleTN, USA; ^3^Department of Biostatistics, Vanderbilt University Medical Center, NashvilleTN, USA

**Keywords:** flow cytometry, Neubauer chamber, nuclear suspension, cell counting

## Abstract

The large size of primate brains is an impediment to obtaining high-resolution cell number maps of the cortex in humans and non-human primates. We present a rapid, flow cytometry-based cell counting method that can be used to estimate cell numbers from homogenized brain tissue samples comprising the entire cortical sheet. The new method, called the flow fractionator, is based on the isotropic fractionator (IF) method (Herculano-Houzel and Lent, 2005), but substitutes flow cytometry analysis for manual, microscope analysis using a Neubauer counting chamber. We show that our flow cytometry-based method for total cell estimation in homogenized brain tissue provides comparable data to that obtained using a counting chamber on a microscope. The advantages of the flow fractionator over existing methods are improved precision of cell number estimates and improved speed of analysis.

## Introduction

Cell numbers vary across areas and regions of the cerebral cortex in primates, however, there are currently only low-resolution, incomplete or no cell number maps available for some primate species, including humans (Collins et al., 2010b). Detailed, high-resolution cell number maps, in large-brained primate species like the human will require cell counts in thousands of small cortical samples to more fully characterize the total cell numbers in identifiable cortical areas and regions and overall patterns of cell distribution (Collins, 2011).

The most commonly used methods for cell number estimation in the brain are stereological methods. Stereological methods can be used to estimate the total number of cells, their shape, size, and volume in sectioned and stained tissue (e.g., Schmitz and Hof, 2005). These methods are best suited to cell population estimates of small, homogeneous brain areas with well-defined boundaries and are less practical for producing detailed cell number maps across the whole cortex from large brains.

As an alternative, but not a replacement for stereological methods, the isotropic fractionator (IF) method (Herculano-Houzel and Lent, 2005) was developed to provide reproducible estimates of total numbers of cells in a shorter period of time. The method relies on the assumption that every cell in the brain contains one nucleus. Brain samples are mechanically dissociated into a homogeneous suspension of cell nuclei, eliminating the problem of heterogeneity in cell distribution in tissue sections. Using manual counting on a Neubauer counting chamber and a fluorescence microscope, the number of nuclei is estimated in a small aliquot from the nuclear suspension, and the data are extrapolated to the total suspension volume using the published formula. The total number of cells contained within a tissue sample can be estimated with this method in hours. The IF method, as originally presented, then goes on to take a separate aliquot from the nuclear suspension that is processed for an immunocytochemical marker that preferentially labels neuronal nuclei, the anti-NeuN antibody. A second count of the antibody-tagged aliquot from the suspension is used to determine the proportion of total cells that are neurons. When both counts are complete, the percent neurons is multiplied by the total cells to estimate the total number of neurons in the suspension and by subtracting the total neurons from the total cells, the number of non-neurons is estimated. It is therefore imperative that the total cell population be accurately estimated, because accurate estimation of all other cell populations depends on an accurate total cell estimate. The present report is limited to a presentation of a flow cytometry-based method for estimating total cell numbers. The IF is well-suited to cellular analysis of easily dissectable brain structures and only becomes cumbersome when the number of samples to be analyzed is very large.

Here we present a new method, the flow fractionator, that is based on the IF method, but uses flow cytometry to count the total numbers of cells in a homogenized suspension, rather than manual counting on a fluorescence microscope. The total number of cells in a nuclear suspension is estimated using DNA staining with 4′,6-diamidino-2-phenylindole (DAPI) and Countbright absolute counting beads (Invitrogen). We compare manual microscope counts obtained using the IF to flow cytometer cell counts on the same subset of samples from two different baboon cortices to test the accuracy of the flow cytometry estimates relative to the microscope-based counting method. A fairly detailed dissection of the cortex from a human brain results in at least 800–1000 tissue samples per cortical hemisphere, depending on the size of the brain and the size of the dissected pieces. A finely dissected cortex from an adult baboon brain can result in 400–500 tissue samples per hemisphere, so this species provides a good test of the feasibility of accurate, high-throughput cell population estimation that may be applicable to even larger brains, such as the human brain. Cell population estimates from the IF procedures have been compared to estimates based on stereological and other cell quantification methods and have been found to be comparable (Tsai et al., 2009). Thus, in this report, we compare the results of our flow fractionator method to comparable IF counts on a subset of the same samples of cortical tissue.

## Materials and methods

### Sample preparation

**Figure 1:**
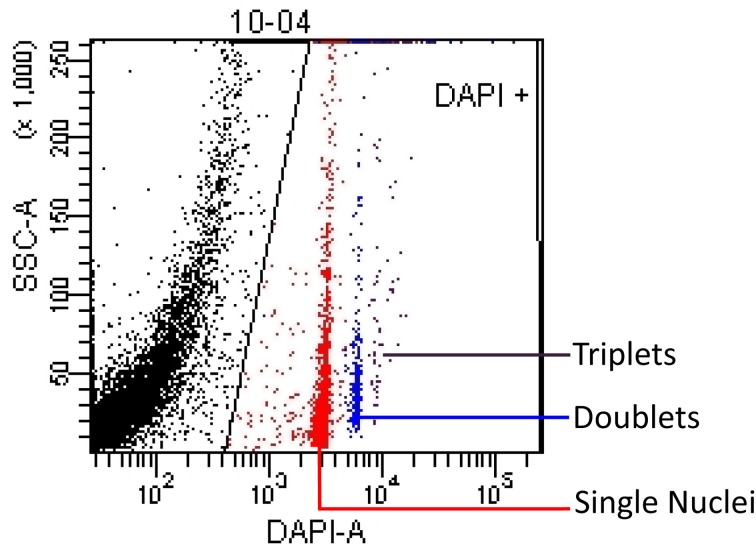
**Nuclei events are shown on a SSC-A (Side Scatter Area) vs. DAPI-A scatterplot.** A polygon gate was used to select the nuclei based on DAPI expression. This analysis profile allowed us to quantify the concentration of singlet (red), doublet (blue) and triplet (purple) nuclei in a known volume of sample. The black area on the left edge of the plot contains debris that is excluded by the nuclei gate.

The brains from two baboons (case 11-31 and case 10-04) (*Papio hamadryas anubis*) were obtained from the tissue distribution program at the Texas Biomedical Research Institute. The baboons were both 14-year-old adult females. The brains were perfused with 0.9% phosphate buffered saline (PBS) and shipped overnight in the same solution. Upon arrival, the brains were bisected and one cortical hemisphere was separated from the subcortical structures, the pia was removed, and the sulci were opened to flatten the cortical sheet. The flattened cortex was viewed on a light box. The primary visual cortex (V1) was readily identified and dissected from the rest of the flat cortex. The visual area MT, primary auditory cortex (A1) and primary sensory areas were identified by their dense myelination. A photograph was taken of the flattened cortex, and the identifiable cortical areas were drawn onto the photograph and dissected from the cortical sheet using a scalpel. The entire cortex was post-fixed in 2% paraformaldehyde. The hemispheres were later dissected into 196 pieces (11–31) and 177 pieces (10-04) that were approximately 5 × 5 mm in size. Cortex was cut into strips first, then the strips were cut into smaller cubes. All cuts are made with a scalpel. Each tissue piece was numbered and assigned to a cortical area, if possible. Assigned sample numbers began at the caudal end of the flat cortex, and ascended rostrally. Each tissue piece was weighed and the map of cut pieces was photographed so the surface area of each piece could be determined. Each dissected tissue piece was processed as an individual sample. Every sixth sample was evaluated with both counting methods. Therefore, samples from multiple cortical areas, distributed evenly across the cortical extent, were used. Details of tissue processing methods appear in a prior publication, and photographs of tissue cube maps appear in Figures 1 and 3 of Collins et al., 2010b. Briefly, cortical tissue pieces were homogenized using a glass Tenbroeck tissue grinder (Fisher Scientific) and a dissociation solution of sodium citrate and triton X-100 in distilled water. The resulting homogenized suspensions contained free-floating nuclei. The total suspension volumes were determined based on the sample density, resulting in suspension volumes between 2 ml and 6 ml. Nuclei were stained with DAPI for both flow cytometry (0.5 mg in 200 ml PBS) and microscopic (2.0 mg in 200 ml PBS) analysis. DAPI binds strongly to DNA and labels all nuclei in the suspension, regardless of cell type. DAPI fluoresces bright blue with ultraviolet excitation (460 nm emission), which is ideal for nuclei counts using both flow cytometry and manual microscope counting methods. The flow cytometry concentration of DAPI is one fourth that used for the microscope, due to the increased sensitivity of the laser detection.

### Method 1: Neubauer chamber nuclei counts

Free-floating, DAPI-stained nuclei from the main suspension samples were counted to estimate total cells using fluorescence microscopy and a glass Neubauer counting chamber and matched coverslip. The main suspension samples were well-mixed, and a 0.5 ml aliquot of the homogenized main suspension was spun down and re-suspended in a mixture of 0.1 M PBS and DAPI. The microscopist that counted the DAPI positive nuclei was blind to the position of the sample in the cortex and also did not have access to the flow cytometry data. The same expert microscopist (NAY) did all of the microscope counts to avoid inter-counter variation, though, in general, the consistency of data across counters is excellent. Several samples were counted three times to assess the reliability of counts taken from the same sample, and any variation associated with loading the Neubauer chamber (11 μl/chamber load; minimum of 44 μl). Several samples were also evaluated multiple times from the same main suspension to assess experimenter error in sampling from the main suspension. All suspensions were vortexed well prior to chamber loading.

### Method 2: flow fractionator nuclei counts

The main suspensions were thoroughly mixed prior to sampling for flow cytometry. A 50 μl aliquot from the main nuclear suspension was added to a 250 μl mixture of PBS and DAPI. A fixed volume of 50 μl of Countbright absolute counting beads (Invitrogen) was added to the sample prior to evaluation using a Becton Dickson (BD) 5-laser LSR II flow cytometer equipped with a 355 nm laser. Each sample was vortexed prior to running on the LSR II, and evaluated for the numbers of nuclei events that occur coincident with 1000 bead events. All samples were prepared in duplicate to assess variation associated with sample preparation and to detect sub-optimal suspension preparation. Two separately prepared samples from each main suspension result in two separate measures and allow us to calculate a coefficient of variation for each main sample to detect samples with variable results that may need to be more closely examined or re-run. Several of the same samples were also repeatedly measured to assess variation associated with the flow cytometer instrumentation. Two-parameter interrogation of Side Scatter (SSC-A) and DAPI (DAPI-A) was used to place a selection gate around the DAPI positive nuclei for quantification. Because the forward scatter (FSC-A) and side scatter attributes are associated with nuclei size and internal complexity, respectively, the DAPI-stained subset was shown on a SSC-A vs FSC-A scatterplot; nuclei events appeared to be approximately 5–15 μm in size. Every attempt was made to include as many nuclei as possible in the nuclei gate, while avoiding the small debris in the samples. The flow cytometry expert (DKF) making decisions about gating was blind to the sample attributes and to the data collected on the microscope. All flow cytometry experiments were conducted in the Vanderbilt University Medical Center Flow Cytometry Shared Resource.

### Flow cytometry acquisition and analysis

All samples were analyzed on a BD™ LSRII instrument using BD™ FACSDiva v. 6.1.3 software. The DAPI-stained nuclei were excited using an Xcyte 355 nm laser. The photons were segregated using a 450/50 bandpass filter. It was determined that when using our configuration, the CountBright™ Absolute Counting Beads (Invitrogen) exhibited the greatest signal to noise ratio using a Compass™ 315 M Diode Pumped laser that emits photons at 532 nm (Coherent) for excitation and collects the emitted photons from the CountBright™ beads using a 576/26 bandpass filter. Due to the broad excitation spectra of the beads, it was necessary to develop a gating scheme that could discriminate beads from nuclei and cellular debris.

### Flow cytometry gating and total cell calculation

FACSDiva software was used to reduce the spectral overlap properties between the CountBright™ beads and the DAPI-stained nuclei. A scatterplot was made showing SSC-A (Side Scatter Area) vs Beads (not shown) and a rectangle gate labeled “beads” was used to identify the bead population on the basis of fluorescence. All of the events not falling within the “beads” gate were shown on a SSC-A (Side Scatter Area) vs. DAPI-A gate scatterplot (Figure 1). Nuclei were gated based on DAPI expression. Due to the autofluorescent properties of the nuclei, which intensify with increasing internal complexity, a polygon gate was used to select the nuclei with a SSC-A vs. DAPI-A scatterplot. This analysis profile allowed us to quantify the number of nuclei in a known volume of sample that we could extrapolate to the total volume of our suspension, thus giving us an absolute number of nuclei per sample of brain tissue.

**Figure 2:**
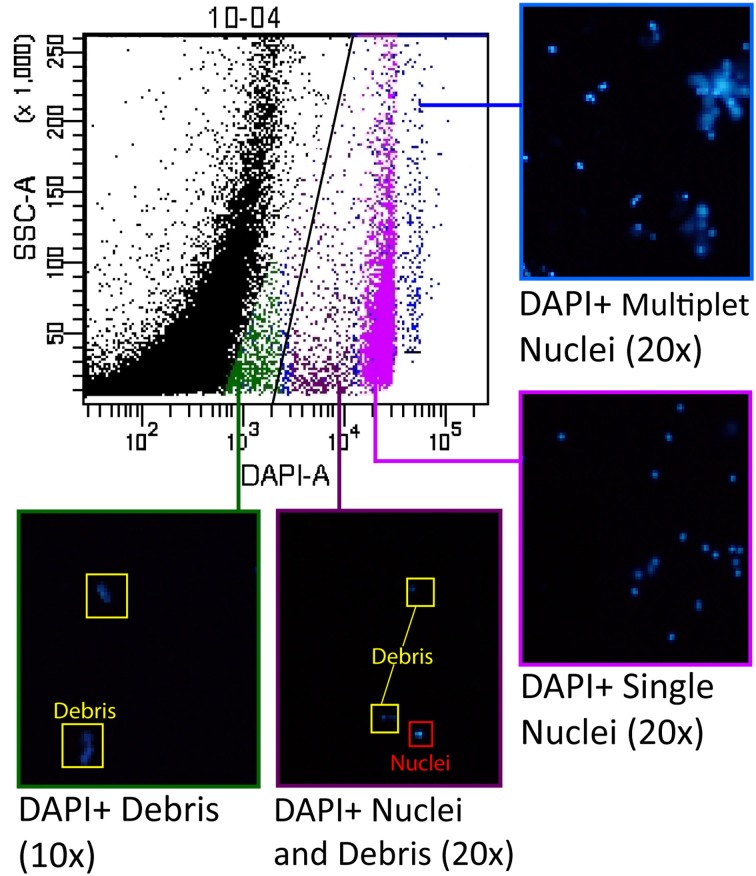
**Groups of nuclei were selected from the scatterplot for analysis by cell sorting to ensure optimal nuclei gate placement.** Groups of nuclei resulting from the sort were examined under a fluorescence microscope to identify the characteristics of each of the major subpopulations visible on the scatterplot. The area of the scatterplot indicated in blue contained primarily multiplets of nuclei (“doublets”, “triplets”, etc.), while the area indicated in pink contained the DAPI positive “singlet” nuclei that were brightly labeled. The area of the scatterplot indicated in purple contained some debris, but also a population of dimly labeled and irregularly-shaped nuclei. The scatterplot area indicated in green contained only debris.

To optimize the separation between debris and nuclei, careful gating techniques were used to identify the auto-fluorescent properties of the debris and ensure that the majority of nuclei are counted while excluding debris from the sample. We determined the fluorescent profile for all channels analyzed based on the initial DAPI postive gate, and identified the unknown profile as the debris. To further confirm that we were segregating nuclei and debris in our gating scheme, we sorted the different populations present on a SSC-A vs. DAPI-A scatterplot using a BD FACSAria III. Once the nuclei and/or debris were sorted, we examined the results under a fluorescence microscope to positively identify the contents of particular zones on the scatterplot (Figure 2).

While using FACS analysis to calculate absolute numbers of events, it was necessary to account for aggregating cells or nuclei. The assumption was made that the samples had been brought to a single nuclei suspension and each nucleus would pass through the lasers one at a time. With proper sample preparation, the vast majority of the events processed will be single events, however, there will always be a percentage of events that are aggregates, or processed in a contiguous manner, resulting in a single event that has the fluorescent and scattering properties of roughly twice a single cell event (see Shapiro, 2003). Therefore, total nuclei counts performed on a flow cytometer can only be accurate if “doublets” are taken into account. To identify doublets, we measured the mean fluorescence intensity (MFI) of the “singlets” and compared it to MFI of the doublets. The doublet sub-population had a MFI value that was roughly twice that of the singlet sub-population and possessed a different voltage pulse height and width ratio of the FSC-A and side scatter signal from a singlet event with equal size and internal complexity (Shapiro, 2003). Plots of FSC-A height vs. width were displayed with singlets and doublets, and the populations clearly display different ratios of height and width pulse geometry, clearly identifying the doublet populations as containing more than one nuclei, therefore we counted this population twice.

When the final gating was done on a duplicate set of the samples from a single case, the nuclei included in each gate were summed and the numbers converted according to the guidelines for using the Countbright beads (Invitrogen). Nuclei number was determined for each sample by calculating the nuclei/μl by multiplying the number of cell events/the number of bead events and the assigned bead count/volume of the suspension aliquot. The main suspension volume was multiplied by the nuclear concentration to determine the number of nucleus in the main suspension.

### Statistical analysis

Our goals included (1) determining the repeatability of counts done by traditional IF methods (hemocytometer, microscope, and human counter) or by flow cytometry, as well as (2) determining the concordance between these two methods. To determine repeatability, at least two aliquots from the same samples were measured using a given method (32 samples from baboon case 11-31 and 28 samples from baboon case 10-04. Every 6th sample from each case was tested.), and the intraclass correlation was calculated using random effects analysis of variance. The intraclass correlation varies from 0 to 1 (0–100%) where a 1 indicates perfect repeatability within a given sample and a 0 indicates inability to distinguish samples. In addition, for the IF method, we quantified the relative sources of variation between samples, sample aliquots, and repeat aliquot counts, using a REML model with random effects (Marchenko, 2006). This latter analysis asked whether there was significant variation in the preparation of multiple aliquots versus counting the same aliquot. For these calculations, we used *xtmixed* and *loneway* commands in Stata statistical software v.12.1 (www.stata.com).

To determine concordance between methods, Lin's concordance correlation was calculated from counts on two baboon cortical hemispheres (Lin, 1989, 2000). Lin's correlation is more appropriate to compare paired measures than is Pearson's correlation, because it takes into account both precision in the data (tightness of the data about its reduced major axis) and accuracy (closeness to the 45 degree line representing perfect concordance in a scatterplot of the paired data). To determine Lin's correlation and the reduced major axis fit of flow cytometry and IF counts, we used a Stata package called *concord* (see Steichen and Cox, 1998). This package also provides an *F*-test of equal means and variances for the methods being compared (Bradley and Blackwood, 1989).

## Results

The flow fractionator method resulted in repeatable cell population estimates that were in excellent agreement with data collected on the microscope using the IF method.

**Figure 3:**
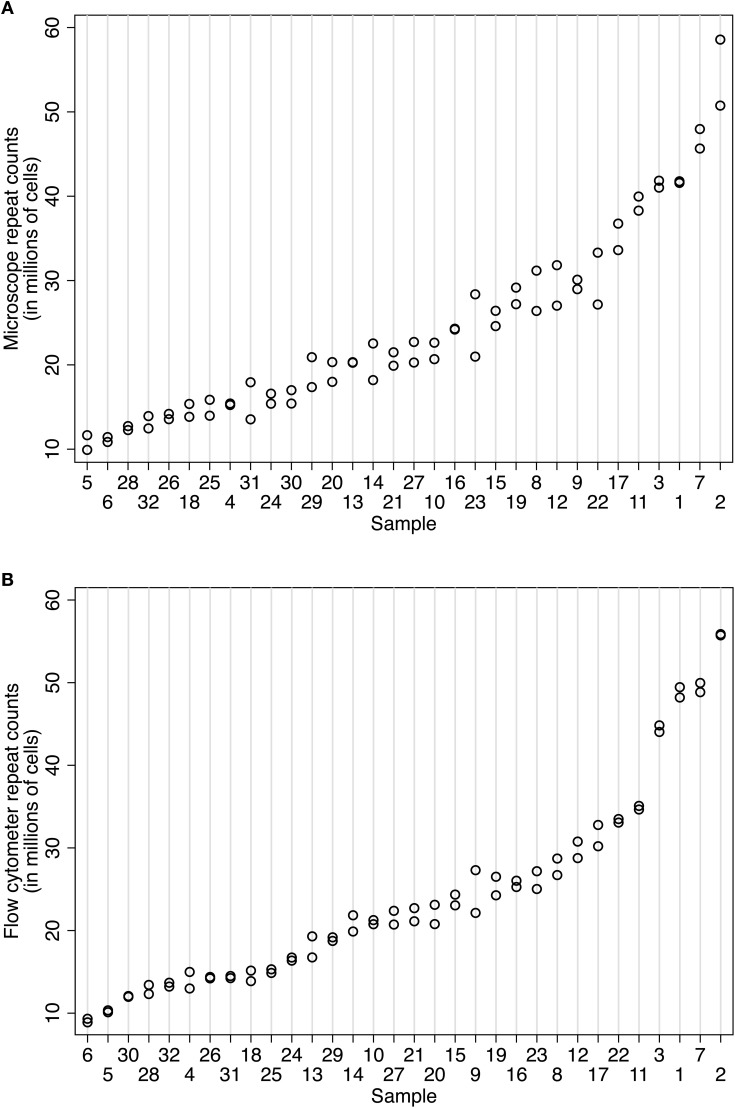
**Comparison of repeatability measures using (A) the isotropic fractionator and (B) the flow fractionator.** In both panels, the samples are organized along the x-axis by increasing mean cell density. The number of nuclei counted in a sample is indicated on the y-axis. Each count is represented by one circle. The same aliquot was counted at least twice to isolate error associated with repeat counting from the same sample (i.e., counting error). Two sets of 32 samples from case 11-31 were prepared for this repeatability measure. **(A)** Repeated cell estimates using the isotropic fractionator produced an intraclass correlation of 0.96 (95% CI 0.934–0.988). The within-sample standard deviation was 2.2 million cells. **(B)** Repeated cell estimates using the flow fractionator for case 10-04 used two sets of 28 samples and produced an intraclass correlation of 0.99 (95% CI 0.984–0.997) and a within-sample standard deviation of 1.16 million cells.

Counts obtained using the Neubauer chamber or flow cytometer methods were both highly repeatable (Figures 3A and B). Using 32 samples from the baboon case 11-31, our expert human counter (NAY) produced IF counts ranging from 10.78 to 54.65 M cells with an intraclass correlation of 0.96 (95% CI 0.934–0.988). The within-sample standard deviation (SD) was 2.2 million cells, and the average coefficient of variation for microscope counts was 7.22%. On different aliquots from the same 32 samples, the flow cytometer (overseen by DKF) produced counts ranging from 9.10 to 55.79 M cells, with an intraclass correlation of 0.99 (95% CI 0.984–0.997) and a tighter within-sample SD (1.16 million cells) and average coefficient of variation (4.04%). These observations are graphically illustrated in Figures 3A and B. The flow fractionator produced more consistent repeat counts with less variation between samples prepared from the main suspension sample. Using variance components analysis on a smaller number of human cortex samples, we determined that the majority of variation was between sample (92.7%, biological signal), with less due to counts on a given aliquot (7.3%, technical error), and no significant variation between aliquots from the same suspension. In other words, very little technical error accrues from sub-sampling a given stock suspension.

**Figure 4:**
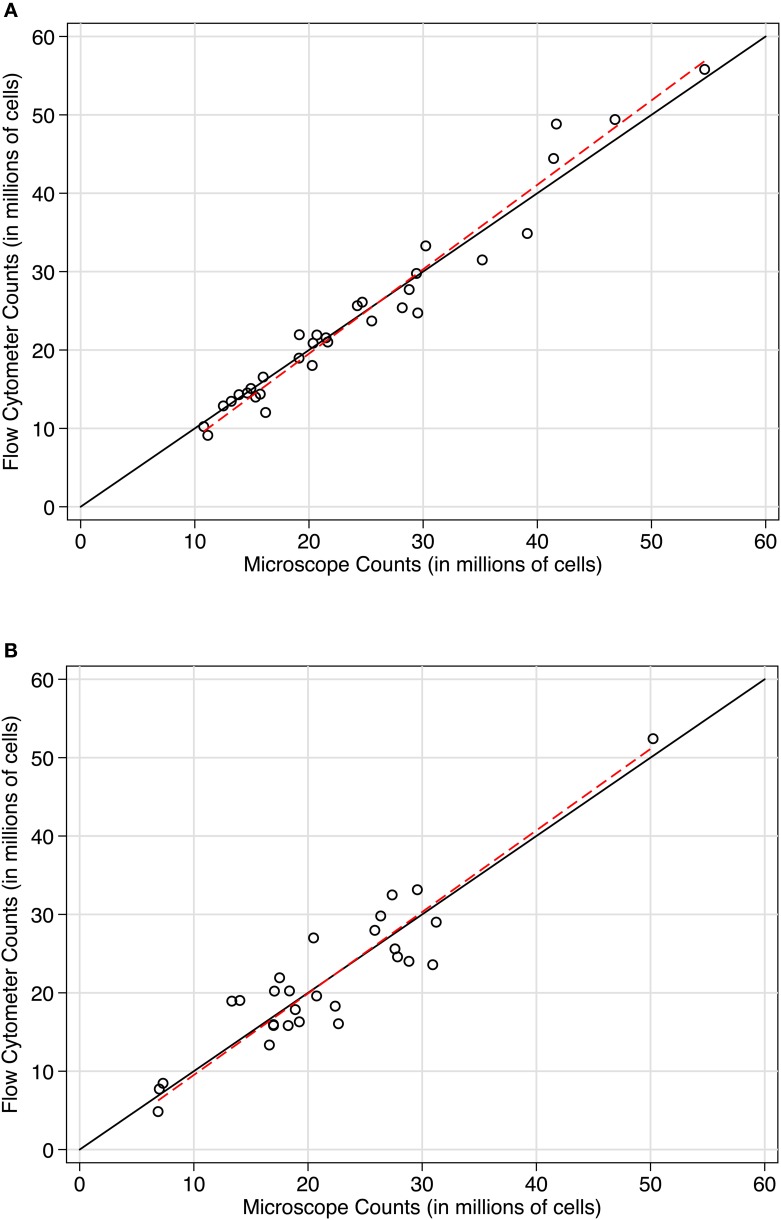
**Concordance scatterplots of cell density estimates in (A) case 11-31 and (B) case 10-04.** Points are plotted with flow fractionator counts along the Y-axis and manual microscope counts on the X-axis. The black line is the line of perfect concordance, Y = X in each plot. The red line represents the linear regression. The concordance between flow fractionator counts and manual counts were **(A)** In case 11-31, Lin's concordance correlation was 0.977 (*n* = 32) and **(B)** In case 10-04, Lin's concordance correlation was 0.915 (*n* = 28).

To directly compare the microscope and flow cytometer estimates of total cell number, we used both approaches on the sample samples. With microscope counts using a Neubauer chamber as the established method, concordance with flow cytometer counts was excellent in two independent baboon hemispheres (see Figures 4A and B). In case 11-31, the Lin's concordance correlation was 0.977 (*n* = 32 paired samples). The reduced major axis slope of the data was close to perfect concordance at 1.077. The average difference between flow cytometer and Neubauer chamber counts was −143,000 cells (SD of the differences = 2.473 million cells), with 95% limits of agreement between −4.989 and 4.703 million cells. Results were similar in case 10-04. Lin's correlation was 0.915 (*n* = 28 paired samples). The reduced major axis slope was 1.04. The average difference between paired measurements was −22,000 cells (SD of the differences = 3.777 million cells) with 95% limits of agreement between −7.425 and 7.380 million cells). In both baboon cases, a Bradley-Blackwood *F*-test did not reveal significant differences in means or variances of the two methods: [case 11:31, *F*_(2, 30)_ = 2.093, *P* = 0.14 and case 10-04, *F*_(2, 26)_ = 0.126, *P* = 0.88]. These statistics confirm the strong correspondence between methods visible in the methods comparison plots shown in Figure 4. In Figures 4A and B, counts using the newer approach (flow fractionator) are plotted on the Y-axis, and counts using the established manual counting approach on the microscope are plotted on the X-axis. If the methods were in complete agreement (100% concordant), the counts would lie along the line of perfect concordance Y = X (black line). The fit of the data by linear regression (red line) closely follows the line of concordance, with very little constant or proportional bias.

## Discussion

Comparisons of total cell estimates in homogenized nuclear suspensions using a flow cytometer (flow fractionator) to those using a Neubauer chamber and fluorescent microscope (isotropic fractionator) show excellent agreement between the two counting methods. The flow fractionator estimates of total cells were more consistent in sample repeatability compared to manual counting, and also produced data in a shorter period of time. This novel methodology makes it possible to process the thousands of samples that would be necessary to create high-resolution cell density map of large primate brains, such as the human brain.

### Manual versus automated cell counts in tissue suspensions

The use of a flow cytometer as described in this paper presents no disadvantages for determination of total cells, relative to standard application of established microscope-based counting methods of a tissue suspension preparation. We expected that the most significant sources of error would be related to sample preparation, as this is where the flow fractionator differs most from the IF, however, the close agreement between nuclei estimates obtained with both counting methods suggests that error in sample preparation does not contribute significantly to the data. Indeed, because counts by the flow cytometer were more repeatable than manual hemocytometer counts at the microscope, and because both methods appeared consistent with each other, we have established counts by the flow cytometer as a viable alternative. Furthermore, as counts on the flow cytometer are much more rapidly obtained, its use improves the efficiency of data collection, particularly for studies in which thousands of samples are to be analyzed. It is possible to obtain counts from 100 samples, in duplicate, in a two-hour period of time using a flow cytometer. Comparable counts on the microscope (200 total) would take more than a week of full-time counting in our hands, with one person counting an average 25 samples per day.

To develop the flow fractionator, we sought to incorporate high-throughput methods for cell number estimation that are increasingly prevalent in medical diagnostics. While manual counting methods are often referred to as the reference method or “gold standard” for estimation of cell content in biomedical diagnostics fields such as blood cell content (see Briggs et al., 2007) and fertility testing (see World Health Organization, 1999), manual methods have largely been replaced by automated instrumentation that can provide rapid and reliable estimates, and the use of flow cytometry is prevalent (see Eustache et al., 2001; Briggs et al., 2007; Perticarari et al., 2007). In light of the gaining dominance of automated cell assessment systems in medical diagnostics for improved speed, accuracy, and efficiency, we sought to combine the advantages of flow cytometry with the efficient homogenization principles of the IF method and to effectively eliminate the bottleneck created by microscopy work.

Previous attempts have been made to automate cell number estimates in neural tissue using flow cytometry. Surchev et al. (2007) used flow cytometry to estimate the number of cells in homogenized cerebellum samples, and reported that their cell estimates were in good agreement with the cell estimates they obtained from manual counting of the same samples using a Neubauer chamber. Despite good agreement between the data obtained by both methods, they reported that both methods required similar effort for sample preparation, but that the sample preparation method they developed for flow cytometry was subject to more experimental error relative to sample preparation for manual counting. In the present study, we have described how our sample preparation method for flow cytometry requires fewer sampling steps, thereby reducing experimental error. Our statistical analysis of the repeatability of both methods shows that cell number estimates obtained with the flow cytometry are more consistent within a sample than those obtained by a single human expert manually counting the same sample on a microscope. The data obtained on the flow cytometer also took significantly less time to obtain (10–30 s/sample) relative to manual counting (15–20 min/sample). The concordance correlation between results from the two methods with each baboon hemisphere were 0.977 and 0.915, which establishes excellent agreement between flow cytometry-based cell counting and manual cell counting using a Neubauer chamber.

### Cell number analysis in dissociated tissue suspensions versus sectioned tissue

While development of suspension-based methods for obtaining cell counts in brain tissue continues, there should be no misconception that these methods are intended to replace traditional stereological counting methods in sectioned and stained tissue. On the contrary, the methods presented here and in previous reports (Collins et al., 2010a) provide an excellent complement to stereological methods in sectioned tissue. The two major advantages of the flow fractionator method and other suspension-based methods are their speed and accuracy estimating total cells and cell populations in brain tissue. The speed of suspension-based methods like the flow fractionator make it particularly well-suited to very large, finely-dissected brains such as those from larger primates, including humans. The flow fractionator is also suited for analysis of a large number of small rodent brains that are compared in gene manipulation experiments or experiments examining large numbers of inbred strains. These kinds of labor-intensive studies are much better suited to higher-throughput methodology like the flow fractionator. We already know from comparisons with our previously published data from a macaque monkey, that our suspension-based cell estimates are comparable to stereological estimates using the optical fractionator (Christensen et al., 2007; Collins et al., 2010b). The utility of a method like the flow fractionator is clear, but it cannot completely replace section-based methods. The disadvantages associated with the suspension-based methods are that some anatomical information is lost when the small samples of cortex and other brain areas are dissociated into cellular nuclei, just like in RNA and protein analysis preparations, yet valuable data can still be obtained. Cellular morphology can no longer be examined since the cell is reduced to a nucleus, though nuclei can be tagged to identify cell types using previously published methods (Collins et al., 2010a). Cell type information can be determined using markers such as NeuN (anti-neuronal nuclear antigen; e.g., Herculano-Houzel et al., 2008; Collins et al., 2010b), which is specific for neurons, and olig2 (anti-olig2), a transcription factor, which is specifically expressed by oligodendrocytes (Hayashi et al., 2011), among other markers. In a recent paper Duan et al. (2012) examined the spatiotemporal distribution of Pax6-expressing cells in mouse brain sagittal and coronal sections during development and their co-localization with NeuN+ neurons. In the same study, they used the IF method to determine the proportion of Pax6+ cells and NeuN+ neurons, powerfully combining section-based and suspension-based methods to provide a more complete evaluation of cellular structure and localization during brain development in mice. This paper is an excellent example of how rigorous anatomical study can be complemented by strong quantitative data provided by rapid, suspension-based methods.

While stereological methods offer an array of approaches for collecting different kinds of quantitative data, they have disadvantages when applied to very large brains. To completely section and process a human brain in its entirety is costly in time and histological supplies, and given the difficulty of obtaining ideally prepared post-mortem samples, histology may not have a good enough outcome to obtain the highest quality staining, even if the sections are in perfect condition. In smaller, laboratory-perfused and prepared brains and parts of brains, stereological methods are considered the gold standard for quantitative evaluation.

## Conclusions

We have shown here that our flow cytometry-based cell counting method can obtain precise results more rapidly than other available methods, by significantly improving sample throughput in estimating the total number of cells in homogenized neural tissue samples relative to manual counting methods. Counts obtained using the flow fractionators are comparable to those obtained using the IF and manual counting on a microscope. The method detailed in the current paper, in combination with our previous work using flow cytometry to determine neuron and non-neuron numbers (Collins et al., 2010a), makes the flow fractionator the most rapid and efficient method for determining both cell and neuron numbers in brain tissue, and can quickly provide detailed, high-resolution cell and neuron distribution maps in larger-brained primates. The method presented here provides a viable means to evaluate a large number of samples from very large, finely dissected brains in a short period of time.

While stereological- and suspension-based methods both have advantages and disadvantages, we believe that the flow fractionator can provide complementary data to studies using classical stereological methods that will provide powerful neuroanatomical description of primate brains or even for a large number of rodent brains in studies examining many individuals for differences across inbred strains or genetically manipulated animals relative to controls. The production of high-resolution cell and neuron density maps of cortex and other brain structures is feasible for human brains with the higher-throughput flow fractionator methods outlined here. In future studies, it will be possible to combine the use of cell-type-specific markers and the present method for estimating total cells to generate high-resolution cell density maps of cortex in normal brains and in brains affected by aging, neurodegenerative and psychiatric disease.

### Conflict of interest statement

The authors declare that the research was conducted in the absence of any commercial or financial relationships that could be construed as a potential conflict of interest.
